# Platelet-rich fibrin: Basics of biological actions and protocol modifications

**DOI:** 10.1515/med-2021-0259

**Published:** 2021-03-22

**Authors:** Voja Pavlovic, Milan Ciric, Vladimir Jovanovic, Milena Trandafilovic, Predrag Stojanovic

**Affiliations:** Department of Physiology, Medical Faculty University of Nis, Bulevar Dr. Zorana Djindjica, 18000 Nis, Serbia; Department of Traumatology, Orthopedic Clinic, Clinical Centre, Nis, Serbia; Department of Anatomy, Medical Faculty University of Nis, Bulevar Dr. Zorana Djindjica, 18000 Nis, Serbia; Department of Microbiology and Immunology, Medical Faculty University of Nis, Bulevar Dr. Zorana Djindjica 81, 18000 Nis, Serbia

**Keywords:** platelet-rich fibrin, platelets, growth factors, wound healing

## Abstract

Platelet-rich fibrin (PRF) represents second generation of platelet concentrates, which has gained increasing awareness in recent years for regenerative procedures. This biologic additive is completely autologous, easy to prepare, has minimal expense, and possesses prolonged growth factor release, together with several other advantages over traditionally prepared platelet concentrates. Since its introduction, various protocols for PRF preparation have been proposed with different amounts of growth factors and other biomolecules necessary for wound healing. However, reference data about potential effect of some PRF components on hard and soft tissue healing are still conflicting. The current article intends to clarify the relevant advances about physiological role of certain PRF components and to provide insight into the new developmental approach. Also, this review summarizes the evolution of platelet concentrates and biologic properties of different modifications of PRF procedure.

## 1 Introduction

Different platelet concentrates, as surgical adjuvants, have been used to enhance the wound healing and tissue regeneration in modern regenerative medicine. Even the process of wound healing is not precisely determined, and it is well-documented that platelets participate in homeostasis, angiogenesis, inflammation, and tissue regeneration [1]. Platelets, isolated from a peripheral blood, represent an autologous source of more than 1,500 bioactive factors (including growth factors, immune system messengers, and enzymes), which are vital for tissue repair and wound healing [2,3]. The ability of concentrated platelets to provide 6–8 times supraphysiological doses of growth factors [4], represents the basic mechanism, which stimulates wound healing. Also, the growth factors released from platelets stimulate the requirement and differentiation of mesenchymal stem cells and other target cells which are involved in the healing process [3].

Since earlier studies [5,6] demonstrated the ability of several key growth factors, found in platelets, to markedly support tissue regeneration, platelet concentrates have been used more than two decades in regenerative medicine [7]. Platelet-rich plasma (PRP) is an autologous concentration of human platelets in a small volume of plasma centrifuged to reach the supraphysiological concentrations of growth factors [4,5]. The PRP protocol requires multistep centrifugation, addition of nonautologous anticoagulants, and additional use of bovine thrombin or calcium chloride. Also, the PRP technique is mainly focused to achieve high concentration of platelets and growth factors and aims to eliminate leucocytes from final composition [8]. However, the ability of anticoagulants to negatively impact the healing process by preventing coagulation and fibrin clot formation [7] led to the evolution of next generation platelet concentrate. Therefore, in 2001, Choukroun et al., as reviewed earlier [9], introduced a new platelet concentrate termed platelet-rich fibrin (PRF). This novel formulation is completely autologous, prepared without any anticoagulants, and contains high concentrations of host immune cells [10].

The present article intends to summarize the current relevant advances of PRF in tissue repair, and the role of some PRF components in wound healing, and to provide the basic characteristics of different modifications of PRF technique and their possible application in regenerative medicine.

## 2 Brief history of various platelet concentrates

The use of different blood-derived products to stimulate the healing process started more than 40 years ago. In the beginning, platelet concentrates were used only in severe thrombopenia to prevent hemorrhages. Increased application of platelet concentrates for the regeneration of hard and soft tissues was due to the adhesive properties of fibrin matrix, as the end product of coagulation cascade, and the huge amounts of growth factors stored inside the platelets [1]. Initially, the development of platelet concentrates to stimulate tissue repair started with fibrin sealant or fibrin glue. This first bioactive surgical additive is produced by using donor plasma, together with the addition of thrombin and calcium to initiate the polymerization process. These adhesives were originally prepared by donor plasma but can also be obtained from the patient (autologously) or commercially [11]. However, due to the risk of disease transmission, cost of their production, and different concentrations of fibrinogen in plasma, the application of fibrin adhesives in modern regenerative medicine is limited [12,13].

Therefore, during last two decades, autologous blood products with high platelet concentrations such as PRP have been developed to replace the fibrin sealants and improve the healing process. PRP represents first generation of platelet concentrate and was introduced for the first time in 1998 [5]. The functional properties of PRP are mainly based on combining the effects of growth factors, actively secreted by platelets, with fibrin glue properties which results with enhanced tissue healing and regeneration. Even though the PRP has been intensively used for a long time, there is a lack of uniformity in PRP preparation protocol. Up to now, there are more than 40 different systems for PRP production from autologous whole blood [2]. In most of the available protocols, 20–80 mL venous blood are collected from the patients and placed in a tube with anticoagulant to avoid platelet activation and degranulation. Generally, the protocol for PRP extraction can be divided into two parts, including centrifugation and activation. During the first step, two centrifugations are recommended which are usually completed within an hour [13]. First centrifugation leads to blood separation in three distinct layers, by using different density gradients. Buffy coat of white blood cells is formed above the erythrocytes layer (which is at the bottom of the centrifuge tube) while platelets are located just above the buffy coat. Buffy coat and plasma are aspirated, pulled, and transferred to another centrifuge tube (without anticoagulant) for second centrifugation (hard spin). The final centrifugation step concentrates platelets at the bottom of the tube, from where it is very easy to evacuate concentrated platelets in plasma suspension. Finally, the obtained PRP is mixed with activators (thrombin and calcium chloride) at the time of the application [1,11]. The used activators induce degranulation of the platelets and fibrin polymerization, resulting in the formation of platelet gel and realiziation of various growth factors.

In humans, normal platelet count varies between 1,50,000 and 3,50,000 cells/µL of blood. The total number of platelets in the final PRP mainly depends on the protocol used for PRP preparation, and it ranges 2–5 times or more than the physiological level. Usually the PRP blood clot contains 95% platelets, 4% erythrocytes, and 1% white blood cells [14]. Concentrated platelets are responsible for active secretion of growth factors and inducing requirement, proliferation, and differentiation of various cells involved in the regeneration process. On the other hand, if the finally obtained platelet concentrate has reduced platelet amount, PRP will lack its therapeutic effect [15]. Once produced, the autologous PRP is stable for 8 hours and eliminates any concerns over transmissible diseases and immunogenic reactions [5]. Following the PRP activation, the platelets start to release growth factors within the first 10 minutes. Majority of the growth factors (95%) are secreted within the first hour after PRP activation, suggesting that PRP has to be administrated in the first 10 minutes after activation [16]. However, during the last decade, several drawbacks of PRP have been reported. Namely, PRP preparation protocol includes anticoagulant addition that interferes with the natural healing process by preventing coagulation and fibrin clot formation [4,17]. Reaction to bovine thrombin and antibodies to bovine factor *V*_a_ may produce coagulopathies and bleeding disorders after PRP application [11,18]. The releasing of growth factors in a very short period of time as well as the absence of uniformity in PRP preparation protocol led to the formation of new platelet concentrate that will be able to overcome the stated limitations.

Due to legal restrictions about replantation of blood-derived products, PRF was first introduced in France by Choukroun et al. in 2001 [19,20]. This second generation of platelet concentrate has been defined as an autologous platelet and leukocyte-rich fibrin biomaterial, which intends to accumulate platelets, immunity promoters, and released cytokines in the fibrin clot [3,20]. Since the PRF preparation protocol does not require any anticoagulants, wound healing cascade is not inhibited by anticoagulants and clot formation occurs naturally. The addition of bovine thrombin, calcium chloride, or other activators is not necessary to obtain PFR, which eliminates the risk associated with the use of bovine thrombin. In contrast to PRP, PRF has several advantages, presented in Table 1, including high concentration of leukocytes which not only act in immune and antibacterial responses but also promote the wound-healing process [21]. The PRF spontaneously forms dense fibrin network that enables slower degradation rate and therefore delayed release of growth factors to the surrounding tissue during wound healing. The release of growth factors from PRF has been reported up to 7 days for the majority of them [22] and longer for certain ones [23]. Moreover, the advantages of PRF over PRP also involve standard protocol production, reduced expense, and a simple method for production [12]. Briefly, blood sample is collected without anticoagulant in 10-mL glass-coated plastic tubes and immediately centrifuged between 2,700 and 3,000 rpm (around 400 g) for 10–12 min. Following the centrifugation, erythrocytes are located at the bottom while platelet-poor plasma (PPP) is positioned in the upper part of the centrifuge tube. PRP clot, which massively entraps platelets, leukocytes, and growth factors, develops between PPP and erythrocytes layer in the middle of the tube. The obtained PRF is easily collected by removing the upper PPP layer [1,9]. The fundamental principle is to permit the platelet activation and fibrin polymerization as it would physiologically. Platelet activation starts immediately upon contact with a wall of the centrifuge tube and leads to the formation of dense fibrin network and usable PRF clot. Therefore, blood collection and transfer to centrifuge tubes have to be accomplished as soon as possible, i.e., maximum within 2 min and 30 s. If this period is prolonged, fibrin polymerizes in a diffuse way and the obtained PRF will not be clinically usable [1,4].

**Table 1:** Advantages and shortcomings of PRF

Advantages of PRF	Shortcomings of PRF
It is completely an autologous product	The success of PRF preparation mainly depends on the speed of blood handling
Minimizes blood manipulation without biochemical handling	PRF membrane should be used immediately, since the structural integrity of PRF modulates over time
It requires no bovine thrombin, since polymerization occurs naturally	Storage of PRF membrane is not possible due to potential bacterial contamination and dehydration
PRF fibrin matrix contains growth factors, leukocytes, and cytokines involved in healing process	Since it is an autologous product, the quantity of PRF is low and it is not possible to be used in general surgery
It shows extended growth factor release compared to other platelet concentrates	
PRF membrane possesses high flexibility and elasticity	
It is inexpensive and involves simple procedure that requires only one centrifugation step	

## 3 Biologic effects of PRF

Analysis of PRF composition reveals that it consists of fibrin clot-enriched with platelets, leukocytes, immune cytokines, and circulating stem cells [10]. Even though the platelets and leukocytes are the main cells responsible for the biologic activity of PRF, fibrin matrix has important role for therapeutic effect of this platelet concentrate [8,12]. The conversion of soluble fibrinogen into insoluble fibrin that polymerizes takes place with available thrombin present in the blood sample. The way of polymerization markedly influences the biologic characteristics of final fibrin matrix. During PRP preparation, application of bovine thrombin and calcium chloride enables sudden fibrin polymerization. In contrast, PRF processing results in slow and natural polymerization of fibrin, due to the physiologic concentrations of thrombin present in the blood sample. Polymerization of fibrin may be organized in two different ways, namely, bilateral and equilateral junctions [12]. High concentrations of thrombin (during PRP preparation) induce the formation of bilateral junctions and the development of rigid fibrin network. This sudden polymerization enables the entrapment of growth factors and cytokines extrinsically in colloidal suspension between the fibrin network and released massively within the first hour. On the other hand, equilateral junctions and flexible fibrin matrix are established in the presence of low thrombin concentrations, during PRF processing [24]. Slow polymerization of fibrin allows increased entrapment of circulating (intrinsic) cytokines in the fibrin matrix. These molecules will be used only when cicatricial matrix remodeling occurs and is released slowly, providing the long-term effect of cytokines at the injury site [9,12].

Platelets, as the dominant component of PRF, represent the main cells responsible for biologic activity of PRF. Even though these cells have crucial role in blood clot formation, they contain various platelet-derived protein molecules that are involved in wound-healing signaling cascade [21]. All these substances are stored by three types of granules (alpha, delta, and lambda) located inside the platelets. Alpha granules are the main reservoirs of growth factors and the most abundant platelet granule. These granules contain various growth factors responsible for the regeneration of soft and hard tissue after injury, which are released through exocytosis when the platelets are activated [1]. Some of the most important growth factors of PRF, presented in Table 2, include transforming growth factor β (TGF-β), platelet-derived growth factor (PDGF), insulin-like growth factor 1 (IGF1), vascular endothelial growth factor (VEGF), and epidermal growth factor (EGF) [12,20]. Moreover, among the growth factors released from platelets, the PRF contains immune cytokines (Table 2) such as interleukin (IL)-1β, IL-6, IL-4, and tumor necrosis factor (TNF)-α [20].

**Table 2:** Certain growth factors and cytokines present in PRF and their function

Transforming growth factor-β (TGF-β)	Stimulates angiogenesis, fibronectin, and collagen production; prevents collagen breakdown; induces fibroblast and immune cells chemotaxis; inhibits osteoclast formation and bone degeneration
Platelet-derived growth factor (PDGF)	Provokes migration and proliferation of mesenchymatous cell lineage; enables angiogenesis, macrophages chemotaxis, and activation; induces TGF-β secretion from macrophages
Insulin growth factor-1 (IGF-1)	Stimulates chemotaxis and activation of osteoblasts and bone formation; induces differentiation and mitogenesis of mesenchymal cells
Vascular endothelial growth factor (VEGF)	Initiates angiogenesis; enhances permeability of the vessels; induces endothelial cell proliferation and migration
Epidermal growth factor (EGF)	Promotes angiogenesis; stimulates proliferation and differentiation of epithelial cells; increases cytokine secretion in epithelial and mesenchymal cells
Interleukin-1β (IL-1β)	Increases expression of adhesive molecules on endothelial cells; stimulates helper T cell, chemotaxis of lymphocytes; activates osteoblasts
Interleukin-6 (IL-6)	Stimulates B-cell differentiation and antibody secretion; induces differentiation of naive T cells in cytotoxic T lymphocytes
Tumor necrosis factor-α (TNF-α)	Induces neutrophil cytotoxicity; stimulates cell survival and proliferation; enhances the remodeling capacities of fibroblasts
Interleukin-4 (IL-4)	Induces B-cell differentiation into plasmocytes, B-cell class switching to IgE, differentiation of naive helper T cells in Th2 cells

TGF-β is a multifunctional cytokine and member of TGF-β superfamily that consists of more than 30 members. Among them are three isoforms (TGF-β1, TGF-β2, and TGF-β3); and TGF-β1 is the most abundant one [20]. The active form of TGF-β1, secreted by activated platelets, stimulates fibroblast chemotaxis and production of fibronectin and collagen and prevents collagen breakdown. In addition, TGF-β1 induces angiogenesis and chemotaxis of the immune cells [25]. Moreover, it enhances osteoblast proliferation and deposition, together with the inhibition of osteoclasts formation and bone degeneration [12].

The PDGF appears to be one of the first growth factors present at the place of injury. It is composed from two subunits (A and B), and three different isoforms exist, including AA, BB, and AB [2]. The PDGF released from the platelets provides migration, proliferation, and survival of mesenchymatous cell lineage [20]. Furthermore, this growth factor enables angiogenesis, macrophage chemotaxis, and activation as well as TGF-β secretion from macrophages [2]. Since each platelet contains around 1,200 molecules of PDGF, abundant concentration of PDGF in PRF may lead to more profound effect on wound healing and bone regeneration [12].

IGF1, or somatomedin C, is a polypeptide hormone massively present in blood circulation but can also be released during platelet degranulation [20]. It stimulates differentiation and mitogenesis of mesenchymal cells [26] and induces survival signals to protect cells from various apoptotic stimuli [20]. In addition, IGF1 stimulates chemotaxis and activation of osteoblasts resulting in enhanced bone formation [2].

Following the tissue injury, VEGF is released by activated platelets and macrophages. VEGF represents main regulatory molecule for angiogenesis-related processes [12] and has a key role in endothelial cell proliferation, migration, and survival [27]. In angiogenesis, factors like IGF-1 and IL-1β have important role, since they upregulate VEGF expression [28]. Previous report demonstrated that PDGF and EGF may markedly increase VEGF secretion [29].

EGF is a protein that belongs to the EFG family of the proteins; and it is secreted by platelets, macrophages, and fibroblasts [29]. It promotes angiogenesis, chemotaxis of endothelial cells, and epithelialization and shortens the healing time [2]. Also, it increases the cytokine secretion by epithelial and mesenchymal cells [30].

IL-1β is a member of a family of 11 cytokines, which initiates the inflammatory response by regulating the expressions of integrins on leukocytes and endothelial cells [31]. The main sources of IL-1β production include macrophages, monocytes, fibroblast, and dendritic cells. This cytokine elevates the expression of adhesive molecules on endothelial cells and increased chemotaxis of phagocytes and lymphocytes at the site of the injury, together with the stimulation of helper T cells [32]. IL-1β, together with TNF-α, activates osteoclasts and inhibits the bone formation [27].

IL-6 is a prominent member of IL-6 family of cytokines and has important role in cell proliferation, migration, survival, and inflammation [33]. It is mainly produced by lymphocytes, fibroblast, epithelial cells, enterocytes, and osteoblasts following their stimulation [34]. Additionally, IL-6 secretion may be stimulated by other pro-inflammatory cytokines, including IL-1 and TNF-α [27]. Among the B-lymphocyte population, IL-6 is able to stimulate the final differentiation of B cells into plasmocytes and markedly enhances the secretion of antibodies from these cells. Further, IL-6 is one of the necessary cytokines to provoke the differentiation of naive T cells in cytotoxic T lymphocytes [27], and it is massively produced during inflammation and remodeling [35].

TNF-α is an important pro-inflammatory cytokine that plays a key role during inflammation and subsequent wound healing [36]. The main reservoirs of this biomolecule are T cells, neutrophils, and macrophages. Also, TNF-α production is regulated by IL-6 and TGF-β [27]. Signaling by TNF-α induces the production of signaling molecules, cell survival, and proliferation and affects epithelial wound healing [37]. This cytokine provokes remodeling capacities of fibroblasts and neutrophil cytotoxicity. Also, TNF-α modulates the expression of other pro-inflammatory cytokines, including IL-1 and IL-6 [27].

IL-4 is a cytokine that induces Th2 differentiation (differentiation of naive helper T cells in Th2 cells). In addition, it stimulates B-cell differentiation into plasmocytes and induces B-cell class switching to IgE [38]. IL-4 has the ability to promote the activation of macrophages in M2 cells. Induced production of M2 cells results in increased IL-10 and TGF-β secretion and finally reduces the intensity of pathological inflammation. Enhanced secretion of M2 cell is closely linked to wound repair and fibrosis [39].

Studies reveal that together with high amounts of platelets and released growth factors, PRF is enriched with leukocytes and immune cytokines, including IL-1β, IL-4, IL-6, and TNF-α as mentioned above [19]. Such phenomenon represents high scientific value since leukocytes are the main drivers of bone and soft tissue regeneration, by releasing lymphogenic factors responsible for the cellular cross talk in tissue regeneration [8]. In line with these results, recent study showed that reducing the relative centrifugation force leads to a significant increase in the total platelet and leukocyte number and amount of the growth factor, indicating that low-speed centrifugation concept results in increased regeneration potential of PRF [8]. Fibrin and fibrin degradation products stimulate neutrophil migration, activation, and release of neutrophil proteases. These neutrophils, at wound site, eliminate contaminating bacteria through the generation of oxygen radicals and enzyme digestion. Furthermore, fibrin interaction with monocytes and macrophages modulates phagocytosis, showing that macrophages have a pivotal role in the transition between wound inflammation and repair [12]. Without leukocytes, the precise cell-to-cell communication for tissue regeneration is not possible, indicating that platelets are not only responsible for tissue regeneration, but they require leukocytes in their capacity toward tissue regeneration process [8].

## 4 Classification of methods for PRF production

The initial protocol for PRF production, introduced by Choukroun et al. in 2001, requires 10 mL of blood sample to be collected without anticoagulant in glass-coated plastic tubes, which is immediately subject to centrifugation at 2,700 rpm (around 400 g) for 12 min. The obtained PRFis usually termed as Choukroun’s PRF or leukocyte and PRF (L-PRF) [13]. However, in the last few years, the PRF protocol underwent several modifications. These protocols led to the formation of various products with different biology and potential uses.

## 5 Advanced PRF

Since it is well-known that high centrifugal forces shift cells to the bottom of the tube, it was proposed that decreased centrifugation speed may prevent cell loss and increase leukocytes number in the PRF matrix. Advanced PRF (A-PRF) was provided by using reduced centrifugal force of 1,500 rpm (230 g) for 14 min and glass-based vacuum tubes [40]. Production of A-PRF may also be obtained by using the same time of centrifugation (14 min) but with a centrifugation speed of 1,300 rpm (200 g), as was suggested later [41]. The obtained A-PRF is richer in the total number of viable cells compared to the L-PRF. Among them, increase in the number of neutrophils, lymphocytes, and platelets was observed [40]. The presence of immune cells influences the differentiation and maturation of macrophages. This may lead to bone and soft tissue regeneration, mainly through the growth factors released from macrophages [40]. In line with these findings, the previous reports documented that macrophages are responsible for osteoblast differentiation, and bone generation is absolutely limited without these cells [42,43]. In addition, the total amount of released growth factors (TGF-β1, VEGF, PDGF, EGF, and IGF1) was markedly higher in A-PRF compared with L-PRF [44]. On the other hand, some previous findings reported decreased amount of growth factors released from A-PRF in contrast to L-PRF, as was reviewed recently [9]. However, despite intensive research, only limited research data are available, and additional studies are necessary to determine the advantages between and limitations of A-PRF and L-PRF.

## 6 Advanced PRF plus

Additional modification of A-PRF protocol resulted in a new formulation termed advanced rich plasma plus (A-PRF+). Taking into account that centrifugation force has direct effect on the amount of the cells trapped inside PRF matrix, investigations tried to reduce the centrifugal time and therefore decrease the total amount of forces that may lead to cell loss. By lowering the centrifugal speed to 1,300 rpm (200 g) and centrifugal time to 8 min, Fujioka-Kobayashi et al. introduced A-PRF+ preparation protocol [41]. Analysis of the obtained A-PRF+ reveals significantly increased level of released growth factors (TGF-β1, VEGF, PDGF, EGF, and IGF1) compared to the A-PRF and L-PRF. Furthermore, A-PRF+ promoted enhanced migration and proliferation of human gingival cells, in contrast to L-PRF [41]. The observed increase in growth factor release might be associated with higher number of leukocytes entrapped in fibrin mesh due to lower centrifugation speed and time [9]. Moreover, exposure of cultured gingival fibroblasts to A-PRF+ resulted in increased collagen1 mRNA levels after 3 and 7 days of cultivation. Having in mind that collagen represents one of the crucial factors during wound healing and remodeling [45], the obtained results indicate regenerative potential of PRF formulations developed with reduced centrifugation speed and time.

## 7 Injectable PRF

The ability of PRF to be obtained only in the gel form, which is not suitable to be injected, represents one of the main PRF limitations, as compared to PRP. Since PRP has the capability to be delivered in liquid form, it can be used alone or in combination with numerous biomaterials in different fields of regenerative medicine. However, recent research provided new protocol for liquid variation of PRF called injectable PRF (i-PRF). This injectable form of PRF is produced by using blood without anticoagulant and centrifuged at 700 rpm (60 g) for 3 min in plastic tubes without any coatings [46]. Plastic tubes used in this protocol do not effectively activate the coagulation process, since they possess hydrophobic surface [47]. Therefore, this procedure allows the separation of blood components in the first few minutes of centrifugation, placing the yellow layer (plasma, clotting factors, and platelets) at the top of the tube. Achieved layer is easily aspirated and ready to be applied in injectable form [46]. The analysis of growth factor release demonstrated higher early release of growth factors whereas PRF showed increased the long-term release of PDGF-AA, PDGF-AB, EGF, and IGF-1 at 10 days. Moreover, i-PRF showed enhanced mRNA levels of TGF-β at 7 days, PDGF at 3 days, and collagen1 expression at both 3 and 7 days when compared to PRP [48]. The obtained results may indicate more intensive biologic effect of PRF over PRP, but this hypothesis requires further analysis.

## 8 Other methods for PRF production

During recent years, different modifications of PRF protocol have been proposed. One of them includes titanium-PRF (T-PRF) which is obtained by centrifuging the blood sample at 2,800 rpm (around 400 g) for 12 min and uses medical grade titanium tubes to provide PRF [49]. Same authors also demonstrate that T-PRF fibrin network cover larger area and it is thicker compared to the L-PRF fibrin network, indicating that T-PRF may last a bit longer in the tissues [49]. Another modification of PRF, termed concentrated growth factors (CGFs), is mainly based on the i-PRF preparation protocol and may represent the variant of the same platelet concentrate. Authors proposed blood sample centrifugation with altered centrifugation speed, ranging from 2,400 to 3,300 rpm and specific time period for acceleration and deceleration in plastic tubes without any coatings, by using specific centrifuge. Both variants have the same principle to separate plasma and platelets at the top of the tube, from where they can be aspirated and used in an injectable form [50]. However, the reference data are limited for the named modifications of PRF protocol. Additional animal and clinical studies are required to optimize the best material used for PRF preparation and, therefore, markedly enhanced the biologic properties of PRF.

## 9 Conclusion

PRF belongs to the new generation of platelet concentrates with new possibilities for enhanced healing and functional recovery. Natural polymerization process of PRF fibrin network allows physiologic architecture of fibrin matrix, which further supports the PRF advantages in the healing procedure. Due to its easy production and low costs, along with it representing completely autologous platelet concentrate, PRF has been successfully used in regenerative medicine. By using different methods, it is possible to get various PRF types, enabling the versatility in the applications of this platelet concentrate. However, platelet and inflammatory effects and their significance of this biomaterial remain to be clarified, and further studies are required to better understand the additional benefits of this second-generation platelet concentrate.
